# Blue light versus green light fundus autofluorescence in normal subjects and in patients with retinochoroidopathy secondary to retinal and uveitic diseases

**DOI:** 10.1186/s12348-018-0167-2

**Published:** 2019-01-08

**Authors:** Millena Gomes Bittencourt, Muhammad Hassan, Muhammad Sohail Halim, Rubbia Afridi, Nam V. Nguyen, Carlos Plaza, Anh N. T. Tran, Mohamed Ibrahim Ahmed, Quan Dong Nguyen, Yasir Jamal Sepah

**Affiliations:** 1 Ocular Imaging Research and Reading Center, Menlo Park, CA USA; 2 Byers Eye Institute, Stanford University, 2370 Watson Court, Suite 200, Palo Alto, CA 94303 USA

**Keywords:** Retinal imaging, Fundus autofluorescence, Autofluorescence imaging, Blue-light autofluorescence, Green-light autofluorescence

## Abstract

**Purpose:**

The aim of this study is to evaluate the differences in the fundus autofluorescence (FAF) signal between the blue light autofluorescence (BAF) from Spectralis® (Heidelberg, CA) and green light autofluorescence (GAF) 200TxTM (OPTOS, UK, in normal subjects and in patients with retinochoroidopathies (RC).

**Methods:**

In this prospective study, FAF was performed using BL (λ = 488 nm) and GL (λ = 532 nm) on normal subjects and patients with RC. The corresponding pairs of BAF and GAF images from both groups were analyzed using Photoshop. The strength of the FAF signal was measured on a gray scale, where optic disc was a standard to indicate absence of AF. In addition, gray values obtained from three identical points (foveal center, and points of hypo and hyper autofluorescence) in the corresponding BAF and GAF images of normal and RC subjects were divided by the optic disc value to calculate autofluorescence signal ratio (*R*). The *R* values at fovea (*R*1), hypoautofluorescent point (*R*2), and hyperautofluorescent point (*R*3) were compared between BAF and GAF modalities, in normal and in RC subjects separately.

**Results:**

One hundred six pairs (106 eyes) of FAF images analyzed (37 pairs: normal and 69 pairs: RC subjects). In normal subjects, the mean *R*1, *R*2, and *R*3 values for BAF were (1.5 ± 0.88, 1.23 ± 0.58, and 4.73 ± 2.85, respectively) and for GAF were (0.78 ± 0.20, 0.78 ± 0.20, and 1.62 ± 0.39, respectively). Similarly, in subjects with RC, the mean *R*1, *R*2, and *R*3 values for BAF were (1.68 ± 1.02, 1.66 ± 1.15, and 7.75 ± 6.82, respectively) and for GAF were (0.95 ± 0.59, 0.79 ± 0.45, and 2.50 ± 1.65, respectively). The mean difference in the *R*1, *R*2, and *R*3 ratios between BAF and GAF in normal and in RC subjects was statistically significant (*p* < 0.001). The strength of the correlation (*r*) between ratios for BAF and GAF was weak or not statistically significant in both normal and RC subjects (*p* > 0.05).

**Conclusion:**

The distribution and intensity of the AF signal differ in BAF and GAF and cannot be used interchangeably. In BAF, optic disc signal is always weaker than in other areas, which was not true for GAF where optic disc signal was stronger than fovea and hypoautofluorescent point in both groups.

## Introduction

In recent years, the study of retinal fundus autofluorescence (FAF) has provided important information regarding the production of retinal fluorophores during physiological aging and in pathological events [1]. Through noninvasive examination techniques, FAF images can map the metabolic status of both retinal pigment epithelium (RPE) and photoreceptor outer segment.

It is well known that the dominant source of FAF signal results from light excitation of the fluorophores in lipofuscin (LF) [2, 3]. LF is physiologically produced within the RPE and reflects its metabolic activity, which is largely determined by the quantity of photoreceptor outer segment renewal [4]. Among the many bisretinoid LF fluorophores, vitamin A-derived pyridinium-bis-retinoid (A2-E) is one of the most studied components. A2E along with other fluorophores are subject to photooxidation and photodegradation with resulting damage to RPE secondary to formation of advanced glycosylated end products, complement activation, detergent like effect on lysosomal membranes, impaired lysosomal activity, and generation of free radicals within the RPE cells [5–8].

The fluorescence emission from these fluorophores has a broad range which peaks at approximately λ610 nm [2, 9]. However, these bisretinoids have different excitation maxima’s in the visible spectrum including λ430 nm (all-trans-retinal dimer), λ439 nm (A2E), λ426 nm (iso-A2E), λ490 nm (A2-dihydropyridine-phosphatifylethanolamine, A2-DHP-PE), and λ510 nm (all-trans-retinal dimer-phosphatidylethanolamine and all-trans-retinal dimer-ethanolamine) [10–13]. It was also noted that as the excitation wavelength increases, the spectral width of emission spectrum decreases [3].

Such wide range of excitation spectra means that the fluorophores responsible for autofluorescence (AF) in the FAF imaging can vary depending on which excitation wavelength is used. The main excitation light more commonly used in commercial devices has been the blue light (BL) (λ488 nm). However, more recently, green light (GL) (λ514 nm and λ532 nm) were introduced for clinical use in commercial confocal scanning laser ophthalmoscopes (cSLO) and adapted ultra-wide-field retinal imaging systems. In addition to potentially exciting different fluorophores, GL is less absorbed by macular pigments compared to BL and enhances LF signal in macula [14].

Despite the proximity of BL and GL within the light spectrum, a potential difference in FAF signal is yet to be determined in images originated by various commercial devices. Thus, a detailed quantification of the blue light AF (BAF) and green light AF (GAF) signal will facilitate precise interpretation of AF signals in a clinical context. The index study is a prospective investigation with the purpose of evaluating the differences in the FAF signal obtained by the BL used in Spectralis® HRA + OCT (Heidelberg Engineering Inc., Vista, CA, USA) and GL used in P200Tx (OPTOS Inc., Dunfermline, Scotland, UK), and to provide a better understanding of the relationships between the measurements obtained from each device.

## Methods

The index study enrolled normal subjects and patients with an established diagnosis of retinochoroidopathy (RC) secondary to uveitis and other retinal diseases who were being followed at a tertiary care ophthalmology clinic. The study was conducted in compliance with the declaration of Helsinki, US Code of Federal Regulations Title-21, and the Harmonized Tripartite Guidelines for Good Clinical Practice (1996). The study was approved by the local Institutional Review Board. A written informed consent was obtained from all participants. All normal subjects underwent fundus examination to document the health of their retina. Any subject with media opacity was excluded from the study.

### Inclusion and exclusion criteria

Patients were included in the study if they met the following criteria: (1) availability of BAF and GAF images of gradable quality, (2) no lesion involving the optic disc which can affect the optic disc fluorescence, and (3) patients with retinochoroidopathy should have lesions within the posterior pole.

### Fundus autofluorescence imaging

After pupillary dilation with topical tropicamide and phenylephrine, BAF images were taken using the Spectralis® HRA + OCT (Heidelberg Engineering Inc., Vista, CA, USA) after subjects were kept in dark room for 30 min. No other imaging was performed on the study subjects prior to fundus autofluorescence (FAF). Spectralis uses an optically pumped solid-state blue laser (λ488 nm) for excitation and uses a barrier filter to capture fundus emissions above λ500 nm of the spectrum. FAF images of a rectangular 30° × 30° field of view were recorded with an ametropic corrector. To improve the signal-to-noise ratio, nine images were aligned and an average image with 768 × 768 pixels was calculated with the Spectralis® HRA + OCT software (Heidelberg Eye Explorer, v4.3; Heidelberg Engineering, Germany). After a gap of 30 min to account for photobleaching, green light FAF (GAF) images were taken using the Optomap-af function in 100^0^ RexMax mode of P200Tx (OPTOS Inc., Dunfermline, Scotland, UK). A single GAF image was acquired using green light (λ532 nm) for excitation and by capturing fundus emissions between λ570 and 780 nm of the spectrum. The central 30° × 30° field was manually obtained using the V2® Vantage software.

### FAF image registration and autofluorescence signal ratio

The pairs of BAF and GAF were co-registered and analyzed using Photoshop (V S5, Adobe Systems Inc., San Jose, CA, US). Only the central 30° × 30° images were used for analysis. The BL and GL images were re-sized to the same pixel/inch rate and aligned based on various retinal landmarks such as retinal vessels. The strength of the FAF signal was defined by the absolute intensity in a gray scale which ranges from 1 to 256. The value 1 is considered the absolute black color possible and 256 the absolute white color possible. By definition, low pixel values (dark) represent low intensities of autofluorescence signal, and high pixel values (bright) represent high intensities of AF signal.

The optic disc AF intensity was used as a standard to indicate the absence of fluorescence, in both BAF and GAF. Multiple corresponding points (3–5) were selected in the optic disc area of both BAF and GAF images and their intensities were averaged. In addition, the optic nerve head served as an index of background noise. Three points of interest were identified in both BAF and GAF images of normal and RC subjects. These points included the foveal center, one point in an area of hypoautofluorescence, and one in an area of hyperautofluorescence. The software ruler and retinal landmarks were used to guarantee the measurement of the same points in both BAF and GAF images.

**Fig. 1:**
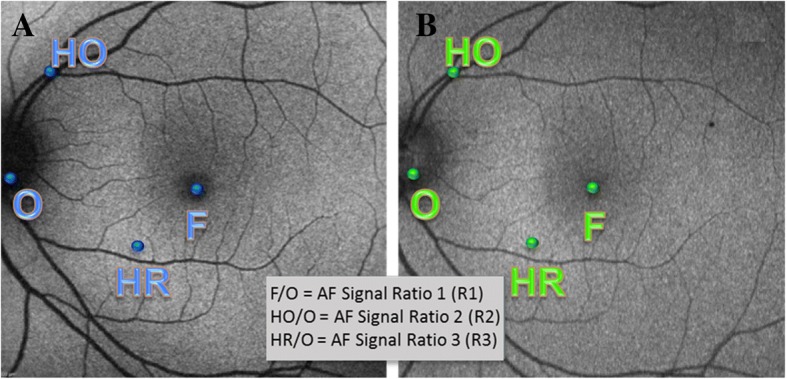
Blue light and green light fundus autofluorescence images with points of interest identified. Fundus autofluorescence images acquired with blue light (**a**) and green light (**b**) in a normal subject showing the identical points of interest measured in both images. For both images, the gray values obtained in the fovea (F), hypoautofluorescent point (HO), and hyperautofluorescent point (HR) were divided from the gray values in the optic disc (O) to calculate the autofluorescence signal ratios *R*1, *R*2, and *R*3 respectively

To compare the AF signal strength between the optic disc and the points of interest, the gray values measured in the three identified points (Foveal center, hypoautofluorescent, and hyperautofluorescent points) were divided from the values in the optic disc (Fig. 1), to compute the AF signal ratio (*R*) in both BAF and GAF images of the normal and RC subjects. The *R* values were calculated and used in this study to account for difference in the two imaging devices in terms of confocality, image capture, and image processing methods. The *R* values were labeled as *R*1, *R*2, and *R*3 for foveal center, hypoautofluorescent, and hyperautofluorescent points, respectively.

In normal subjects, the hypoautofluorescent point selected was in the vessels and the hyperautofluorescent point was chosen in the area 70–150 μm from the fovea. Similarly, in the subjects with RC, a point was selected in the hypoautofluorescent lesion and another point was selected in the hyperautofluorescent lesion.

### Blue light FAF vs green light FAF

The *R*1, *R*2, and *R*3 values were compared to test the agreement and the strength of correlation between BAF and GAF modalities, in both the normal and RC subjects.

### Statistical analysis

The SPSS (IBM® Inc., Chicago, IL) release 19.0.0 was used for statistical analysis. Demographic characteristics of the patients were summarized using descriptive statistics and expressed as mean and standard deviations. The mean *R* values were calculated for points of interest in both BAF and FAF eyes. To test the agreement between *R* values of BAF and GAF at same location, the mean difference and standard deviation were calculated, and Bland-Altman scatter plots were generated. To verify the strength of the correlation, the Pearson coefficients were used.

## Results

A total of 106 pairs (106 eyes) of FAF images were included in this prospective study. Each pair consisted of BAF and GAF images of the same area. Thirty-seven pairs (37 eyes) of the images were from the normal subjects. Seventy-eight pairs (78 eyes) were obtained from subjects with various RCs. Table 1 outlines the demographic characteristics of the study population.

**Table 1:** Demographic characteristics of the study population

Diagnosis	Number of eyes	Mean age years (SD)	Gender (F:M)	Ethnicity	Number of pair of images
Normal subjects with no known ocular disease					
Normal subjects	37 (17 subjects)	32 (± 7)	17 F:14 M	9C: 2AA	37
Retinochoroidopathy secondary to retinal diseases					
High myopia	13	29 (± 6.43)	11 M:2 F	9A: 4C	13
Hydroxychloroquine retinal toxicity	2	38 (N/A)	2 F	2C	2
Aged-related macular degeneration (drusen)	1	74 (NA)	1 M	1C	1
Central serous chorioretinopathy	2	48 (± 8.66)	2 M	2A	2
Cone-rod dystrophy	4	75 (± 0.57)	4 M	2C: 2A	4
Diabetic retinopathy	2	68 (N/A)	2 M	2C	2
Sickle cell retinopathy	1	48 (N/A)	1 F	1AA	1
Lymphoma (intraocular)	1	74 (N/A)	1 M	1C	1
Uveitic retinochoroidopathies					
Panuveitis (idiopathic)	1	30 (N/A)	1 F	1C	1
Vogt-Koyanagi Harada	2	25 (N/A)	2 M	2AA	2
Acute zonal occult outer retinopathy	3	44 (± 21)	3 F	3C	3
Punctate inner choroidopathy	10	31 (± 6.67)	8 F:2 M	10C	10
Birdshot choroidoretinopathy	5	54 (± 4.54)	3 F:2 M	5C	5
Multifocal choroiditis	16	40 (± 8.03)	16 F	12C: 2AA: 2A	16
Sarcoidosis with posterior uveitis	2	83 (N/A)	2 F	2C	2
Serpiginous choroiditis	2	38 (N/A)	2 M	2C	2
Retinochoroiditis of unclear etiology	2	31 (N/A)	2 F	2AA	2
Total	69 (42 patients)	41 (± 16)	38 F:31 M	47C: 15A: 7AA	69

Age: *SD* standard deviation and *N/A* not applicable; gender: *F* female and *M* male; ethnicity: *C* Caucasian, *AA* African-American, and *A* Asian

### Autofluorescence signal ratio for BAF and GAF in normal subjects

**Fig. 2:**
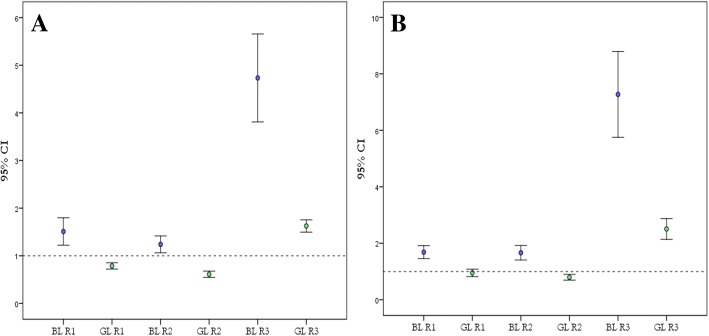
Confidence interval plots. Confidence interval plots for autofluorescence signal ratios (*R*) measured in normal (**a**) and RC (**b**) subjects. The *R* values are represented by blue and green circle for BAF and GAF, respectively. The *R* values above dotted lines represent signals more intense than the optic disc signal (dotted line). Similarly, below the dotted are located the AF signals weaker than the signal in the optic disc

The average *R* in the fovea (*R*1) of normal subjects was 1.5 ± 0.88 and 0.78 ± 0.20 as determined by BAF and GAF, respectively (Fig. 2). Similarly, the average *R*2 in the vessels was 1.23 ± 0.58 and 0.61 ± 0.20 for BAF and GAF images, respectively. Finally, the average *R*3 in the point located 70–150 μm from the fovea was 4.73 ± 2.85 and 1.62 ± 0.39 as assessed by BAF and GAF, respectively (Table 2). Figure 2 plots the 95% confidence interval distribution across for ratios measured by BAF and GAF in the normal subjects.

**Table 2:** Autofluorescence signal ratio (*R*) in normal and RC subjects

Autofluorescence signal ratio (*R*)						
	Blue light (λ488 nm)			Green light (λ532 nm)		
	Mean	SD	95% Confidence interval	Mean	SD	95% Confidence interval
Normal subjects						
*R*1	1.50	0.88	1.26–1.83	0.78	0.20	0.72–0.85
*R*2	1.23	0.54	0.36–2.61	0.61	0.20	0.54–0.67
*R*3	4.73	2.85	3.93–5.76	1.62	0.39	1.50–1.74
RC subjects						
*R*1	1.68	1.02	1.48–1.91	0.95	0.59	0.83–1.09
*R*2	1.66	1.15	1.42–1.93	0.79	0.45	0.70–0.89
*R*3	7.27	6.82	5.98–8.78	2.50	1.65	2.19–2.86

*SD* standard deviation

### Autofluorescence signal ratio for BAF and GAF in RC subjects

In the group of eyes with RCs, the average *R* values followed the same trend seen in normal subjects. The average *R*1 in RC subjects was 1.68 ± 1.02 and 0.95 ± 0.59 as determined by BAF and GAF in the GL FAF images. Similarly, the average *R*2 in point within hypofluorescent lesion was 1.66 ± 1.15 and 0.79 ± 0.45 for BAF and GAF images, respectively. Finally, the average *R*3 in the point located within hyperfluorescent lesion was 7.75 ± 6.82 and 2.50 ± 1.65 as assessed by BAF and GAF, respectively (Table 2). Figure 2 plots the 95% confidence interval distribution across for ratios measured by BAF and GAF in the RC subjects.

### Agreement between BAF and GAF autofluorescence signal ratios

**Fig. 3:**
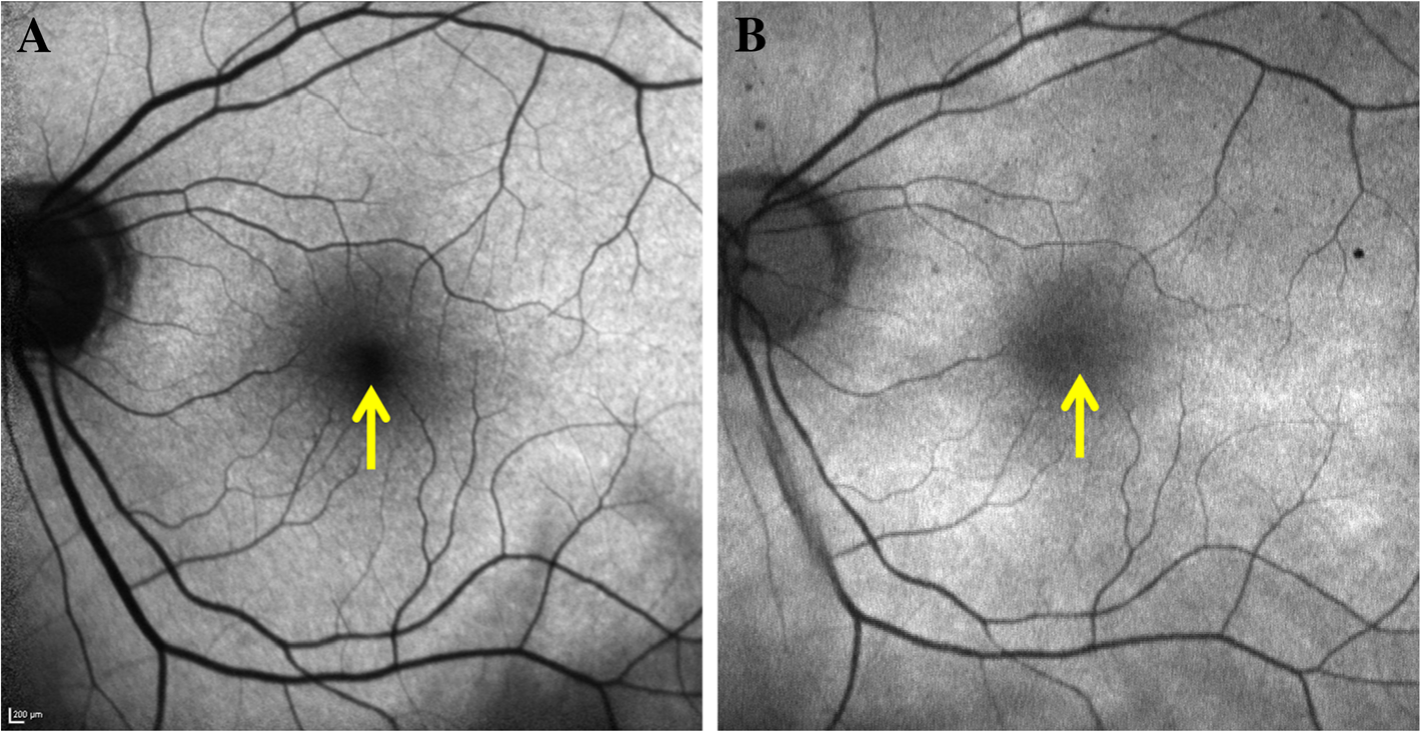
Blue light vs green light autofluorescence signals at the fovea. Blue light autofluorescence (BAF) (**a**) and green light autofluorescence (GAF) (**b**) images at the foveal center demonstrating the differences in the foveal autofluorescence (AF) signal. The AF signal due to BAF (**a**) appears much darker compared to the GAF (**b**) as the blue light is more strongly absorbed by the macular pigments compared to the green light

**Fig. 4:**
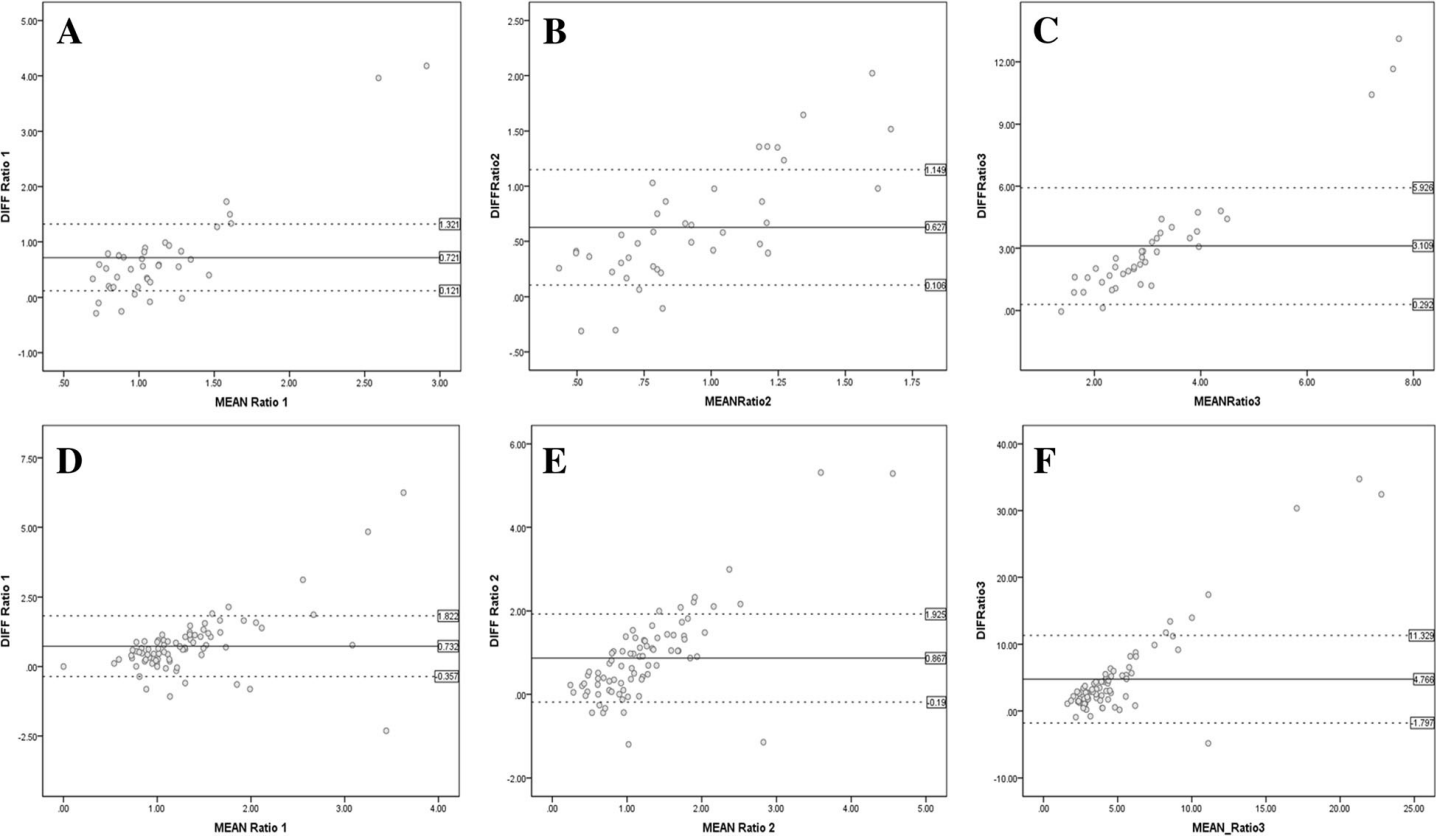
Bland-Altman scatterplots. Bland-Altman scatterplots for differences between BAF and GAF autofluorescence ratios (*R*) in normal (**a** fovea; **b** hypoautofluorescent point; **c** hyperautofluorescent point) and RC (**d** fovea; **e** hypoautofluorescent point; **f** hyperautofluorescent point) subjects

The mean difference in the *R*1, *R*2, and *R*3 ratios between BAF and GAF in normal eyes was statistically significant (*R*1: 0.72 ± 0.9, *p* < 0.0001 (Fig. 3)); *R*2: 0.62 ± 0.52, *p* < 0.0001; *R*3: 3.10 ± 2.81, *p* < 0.0001) (Table 3). Similar results were also observed in eyes with RCs, where difference in the *R*1, *R*2, and *R*3 ratios between BAF and GAF was 0.73 ± 1.09 (*p* < 0.0001), 0.86 ± 1.05 (*p* < 0.0001), and 4.76 ± 6.56 (*p* < 0.0001), respectively (Table 3). The bland-Altman scatterplots for differences between BAF and GAF ratios in normal and RC subjects are shown in Fig. 4. The scatterplots show no agreement between the AF signal ratios of BAF and GAF images, with a statistically significant difference between both groups.

**Table 3:** Difference between autofluorescence signal ratios (*R*) measured by blue light and green light at the fovea (*R*1), hypoautofluorescent point (*R*2), and hyperautofluorescent point (*R*3)

	Mean difference	SD	Sig. (*p*)
Difference between blue and green ratios: normal subjects (*N* = 39 images)			
*R*1 Difference	0.72	0.90	0.000
*R*2 Difference	0.62	0.52	0.000
*R*3 Difference	3.10	2.81	0.000
Difference between blue and green ratios: patients (*N* = 80 images)			
*R*1 Difference	0.73	1.09	0.000
*R*2 Difference	0.86	1.05	0.000
*R*3 Difference	4.76	6.56	0.000

*SD* standard deviation

### Correlation between BAF and FAF autofluorescence signal ratios

The strength of the correlation (*r*) between *R* values for BAF and GAF was weak or not statistically significant in both normal eyes (*R*1 *r* = 0.015, *p* > 0.05; *R*2 *r* = 0.308, *p* > 0.05; and *R*3 *r* = 0.158, *p* > 0.05) and RC eyes (*R*1 *r* = 0.179, *p* > 0.05; *R*2 *r* = 0.407, *p* > 0.05; and *R*3 *r* = 0.276, *p* > 0.05).

## Discussion

With the advent of cSLO, FAF images have left the research laboratories and have become more popular as a propaedeutic tool to assist in the diagnosis, management, and monitoring of certain conditions. However, despite its incontestable importance, FAF image interpretation can be particularly challenging and are subject to individual interpretation of gray intensity and contrast between normal an abnormal area. Many papers have qualitatively characterized the FAF patterns and their progression in various retinal pathologies.

Lois and colleagues were among the first to quantitatively assess the differential distribution of FAF signal in the images obtained with cSLO in normal volunteers and in patients [15]. Their work was primarily based on BL FAF images and did not include other wavelengths. As a first effort to explore the difference between blue and green excitatory lights, Wolf-Schnurrbusch et al. evaluated differences in size of geographic atrophy lesion as assessed by BL (λ488 nm) and GL (λ514 nm) using a modified cSLO (HRAmp; Heidelberg Engineering) [16]. There was a significant difference in measurements of GA lesion size between the two wavelengths. Additionally, GAF was better at delineation of borders of atrophic patches in foveal region and preserved foveal island. Similarly, another study demonstrated slightly larger GA lesion size of GAF compared to BAF with significantly higher intergrader reliability in GAF compared to BAF [17]. Both of these findings were attributed to better contrast offered by the GAF. These findings show that wavelength used to acquire the images may play a role in the visualization of lesions.

Our group proposed a method similar to the one described by Lois et al. to assess the strength of AF signals and to evaluate the relation, in this case ratio, between BL and GL FAF [15]. However, unlike the method of Lois et al., which subtracts the optic nerve head intensity from the retinal signal, our method estimates the ratio between AF signals in various retinal areas and optic disc and does not entail the primary purpose of describing the distribution of the AF in degrees of eccentricity. Additionally, using the *R* values allows us to compare two different imaging devices and account for their differences in terms of confocality, image capture, and image processing methods. In our study, the relation between optic disc and fovea (*R*1) hypo (*R*2) and hyperautofluorescent (*R*3) areas was consistent across normal subjects and subjects with RC within same modality. However, the *R* values were not equivalent across BAF and GAF images. The agreement and the correlation of both FAF modalities were significantly different supporting the findings seen by Wolf-Schnurrbusch and Pfau et al. [16, 17].

**Fig. 5:**
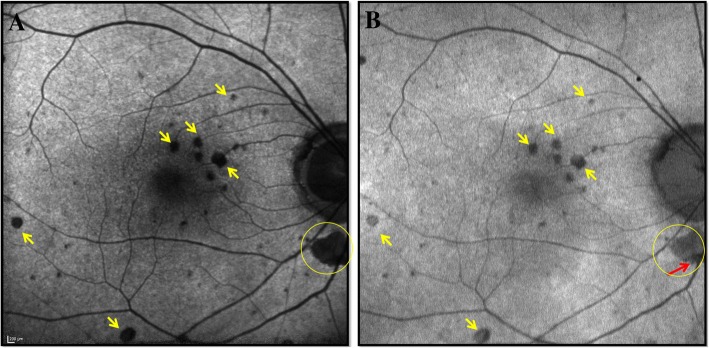
A case outlining discrepancy in blue and green light autofluorescence signals of similar hypoautofluorescent lesions in posterior uveitis. Blue light autofluorescence (BAF) (**a**) and green light autofluorescence (GAF) (**b**) images of an 83-year-old Caucasian woman with posterior uveitis secondary to sarcoidosis (retinochoroiditis), showing hypoautofluorescent lesions (yellow arrows) spread throughout the posterior pole. The fundus autofluorescence images show noticeable discrepancies in the autofluorescence AF signals of the hypofluorescent lesions captured by the BAF (**a**) and GAF (**b**). Additionally, in the lesion inferior to the optic nerve (yellow circle, red arrow), there is a small area of deep loss of AF signal within the hypoautofluorescent lesion as shown by the GAF (**b**). The detail is not revealed by the BAF image

**Fig. 6:**
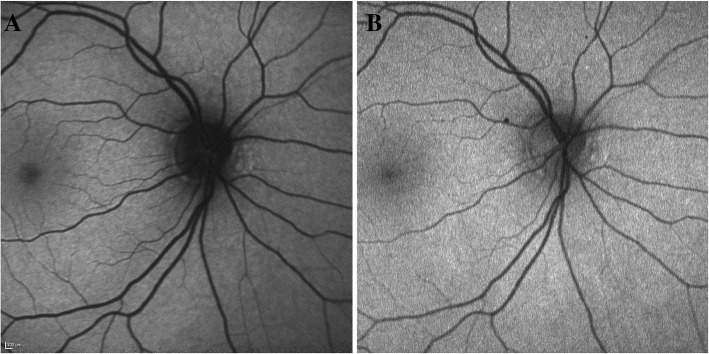
Blue light vs green light autofluorescence signals at the optic disc. Blue light autofluorescence (BAF) (**a**) and green light autofluorescence (GAF) (**b**) images of optic disc. There are noticeable discrepancies in the autofluorescence signal at the optic disc as captured by BAF (**a**) and GAF (**b**)

In BAF imaging of the normal eyes, the gray intensity in the optic nerve head consisted of dark black due to the absence of LF in this area. Similarly, the blood vessels showed a weak strength of the AF signal due to absorption of BL by hemoglobin, yet it was higher than the optic disc. Normally in the BAF, the foveal center appears hypoautofluorescent due to absorption of BL by luteal pigment and melanin. However, the AF signal as detected by our study was still brighter than the optic disc. In GAF imaging of normal eyes, the AF signal at the optic disc was higher than at the foveal center even though normally the AF signal at fovea is higher in GAF due to weaker absorption of GL by macular pigment compared to the foveal AF signal in BAF as BL is stronger absorbed by the macular pigments (Fig. 3) [18]. Similarly, the AF signal at optic disc in GAF was higher than that of the blood vessels. In RC eyes, the *R* values for BAF and GAF signal followed similar pattern to normal eyes. Figure 5 describes a case demonstrating difference in the AF signal of a hypoautofluorescent lesion as captured by BAF and GAF. The optic disc had the lowest AF signal in BAF images compared to fovea and even hypoautofluorescent lesions. On the other hand, optic disc AF signal intensity was higher in GAF images compared to fovea and hypoautofluorescent lesions. The difference in the autofluorescent pattern of the optic disc between BAF and GAF can be because of excitation of different fluorophores by the two wavelengths (Fig. 6). These differences in the AF signals captured by BAF and GAF underscore the importance of not using the FAF images captured by the Heidelberg Spectralis and Optos P200Tx interchangeably.

In our study, we also noted that the range of the confidence interval of the *R* values also differed between BAF and GAF modalities. The intervals were broader in the BAF images compared to the GAF images. Sparrow et al. demonstrated that emission spectra of the fluorophores present in the LF varied depending on the wavelength used [3]. As the wavelength increased, the emission spectral width decreased considerably from 190 nm at λ330 nm to 60 nm at λ545 nm. Therefore, the variance in emission intensities may be responsible to a wider confidence interval of gray scale measurements in BAF noted in our study.

Our study has provided valuable insight about FAF image analysis. A variety of diagnoses, from retinal vascular to uveitic diseases, although small in number, provide a diverse sampling of how BAF and GAF may differ among different entities. However, our study also contained some limitations. The absolute values of the AF signal could not be used for comparison due to the difference in the optics of both devices, translating in different intensity of fundus exposure, gain, and distortions. Also, the sample size of our study was relatively small, especially in specific categories.

## Conclusion

The quantitative evaluation of AF signal is an important step toward the correct interpretation of BAF and GAF images in clinical practice. According to our results, the distribution and intensity of the FAF signal differ in BAF and GAF images acquired by Heidelberg Spectralis® and Optos P200Tx™, respectively. Therefore, the outcomes from these two devices are not simply interchangeable despite using wavelengths which are in close proximity in visual spectrum. The difference was consistent across normal and RC subjects. Further analyses are indicated to confirm if the images from Spectralis® and P200Tx™ may provide similar or different AF characterization of diseases. Moreover, the discrepancies in our findings underscore the importance of evaluating the consequences of design choices made by the manufacturers.

## Abbreviations

AF: Autofluorescence
BAF: Blue-light autofluorescence
BL: Blue light
FAF: Fundus autofluorescence
GAF: Green-light autofluorescence
GL: Green light
LF: Lipofuscin
R: Autofluorescence signal ratio
RC: Retinochoroidopathy
RPE: Retinal pigment epithelium
